# Terahertz Polarization Isolator Using Two-Dimensional Square Lattice Tellurium Rod Array

**DOI:** 10.3390/mi15060745

**Published:** 2024-05-31

**Authors:** Yong Wang, Yanqing Ai, Lin Gan, Jiao Zhou, Yangyang Wang, Wei Wang, Biaogang Xu, Wenlong He, Shiguo Li

**Affiliations:** 1 College of Microelectronics, Shenzhen Institute of Information Technology, Shenzhen 518000, China; 2 Institute of Applied Physics and Materials Engineering, University of Macau, Macau 999078, China; 3 School of Mechanical and Electrical Engineering, Shenzhen Polytechnic University, Shenzhen 518000, China; 4 College of Electronics and Information Technology, Shenzhen University, Shenzhen 518060, China

**Keywords:** terahertz, photonic crystal, polarization isolator, tellurium, complete PBG, 6G

## Abstract

A novel terahertz polarization isolator using a two-dimensional square lattice tellurium rod array is numerically investigated at the interesting band of 0.22 THz in this short paper. The isolator is designed by inserting six hexagonal tellurium rods into a fully polarized photonic crystals waveguide with high efficiency of −0.34 dB. The TE and TM photonic band gaps of the 7 × 16 tellurium photonic crystals are computed based on the plane wave expansion method, which happen to coincide at the normalized frequency domain from 0.3859(*a*/*λ*) to 0.4033(*a*/*λ*), corresponding to the frequency domain from 0.2152 to 0.2249 THz. The operating bandwidth of the tellurium photonic crystals waveguide covers 0.2146 to 0.2247 THz, calculated by the finite element method. The six hexagonal tellurium rods with smaller circumradii of 0.16*a* serve to isolate transverse electric waves and turn a blind eye to transverse magnetic waves. The polarization isolation function and external characteristic curves of the envisaged structure are numerically simulated, which achieves the highest isolation of −33.49 dB at the central frequency of 0.2104 THz and the maximum reflection efficiency of 98.95 percent at the frequency of 0.2141 THz. The designed isolator with a unique function and high performance provides a promising approach for implementing fully polarized THz devices for future 6G communication systems.

## 1. Introduction

Photonic crystal (PhC) devices like circulators [1,2] and isolators [3,4,5] are indispensable in future communications systems, including 6G [6]. In recent years, PhC isolators have attracted much more attention due to their unique performance and integration prospects in microwave photon transceiver modules and optical electronic integrated circuits. In the optical waveband, an integrated magneto-optical isolator was developed by using non-reciprocal phase shift at the 1550 nm waveband [3]. Based on the Faraday rotation of magneto-optical materials, a silicon-integrated optical isolator was demonstrated at the same waveband for the single polarization state [4]. A homogeneously polarized isolator for the TE mode was realized at 1540.5 nm and achieved the experimental isolation of 32 dB [5]. In the microwave band, an integrated isolator with the optimal isolation of 10 dB was designed by using magnetic-free silicon nitride [7]. In our previous work, a 5G magneto-optical isolator and circulator was designed by using an ultra-wideband PhC waveguide in the millimeter waveband [8]. Based on the magnetic resonance absorption effect, the maximum isolation and relative bandwidth were optimized to 49.49 dB and 8.85%, respectively. A wideband microwave photonic circulator depending on the light local effect [9,10] and Faraday rotation effect [11] was numerically investigated by inserting two asymmetric triangular ferrite sheets into a Y-shaped PhC waveguide [12]. The external characteristics of the devices in [11,12] were simulated by the finite element method (FEM) using software called Comsol Multiphysics, which is a numerical analysis technique used to solve complex engineering and scientific problems. It discretizes a continuous problem and divides the solution field into a finite number of small, non-overlapping units, then approximates each unit, and finally combines the solutions of all units to obtain the solution of the whole problem. This method is especially suitable for solving partial differential equations, such as electromagnetic fields and micro-electro-mechanical systems (MEMSs).

PhCs with photonic band gaps (PBGs) also have been used to design multiple devices for photovoltaic applications [13] like filters [14], routers [15], resonators [16], optical logic gates [17], adders [18], phase shifters [19] and some all-optical memory components [20,21,22,23,24,25,26]. The PBGs of some different types of PhCs are generally calculated by the plane wave expansion method (PWEM) based on the Bandsolve module in Rsoftwave, which is a mathematical method used to expand a wave function or physical quantity into a linear combination of plane waves. This method is very useful in calculating the electronic and harmonic structure of solids, because it can consider the lattice periodic structure of solids and describe the band structure of solids more accurately. Based on the Faraday rotation effect, a terahertz (THz) isolator was experimentally demonstrated by using yttrium iron garnet [27]. However, the abundant number of design schemes that include isolators described above are only for a single polarization state of the TE or TM mode.

The aim is to develop a fully polarized isolator to adapt to the requirements of future communications systems. In this work, we focus on realizing a novel polarization isolator by using 2-D tellurium PhCs in the atmospheric communication window of the 0.22 THz waveband, which can isolate the TE or TM mode from mixed modes. Our polarization isolator is designed by inserting six hexagonal tellurium rods into the PhC waveguide with a fully polarized PBG. The TE and TM PBGs of the square lattice tellurium rod array are simulated by PWEM, which happen to coincide with the overlap area. Basically consistent with the frequency domain of the complete PBG, the operating bandwidth of the tellurium PhC waveguide covers 0.2146 to 0.2247 THz. The six hexagonal tellurium rods with smaller circumradii serve to isolate TE waves and have almost no effect on the TM waves. The polarization isolation function and external characteristic curves of the envisaged structure are simulated by the FEM, which can effectively separate the TM from the mixed waves. The designed isolator achieves the highest isolation of −33.49 dB at the central frequency of 0.2104 THz and the maximum reflection efficiency of 98.95 percent at the frequency of 0.2141 THz. Its unique function and high performance provides a promising approach for implementing fully polarized THz devices in future 6G communication systems.

## 2. Composition of the Polarization Isolator

**Figure 1:**
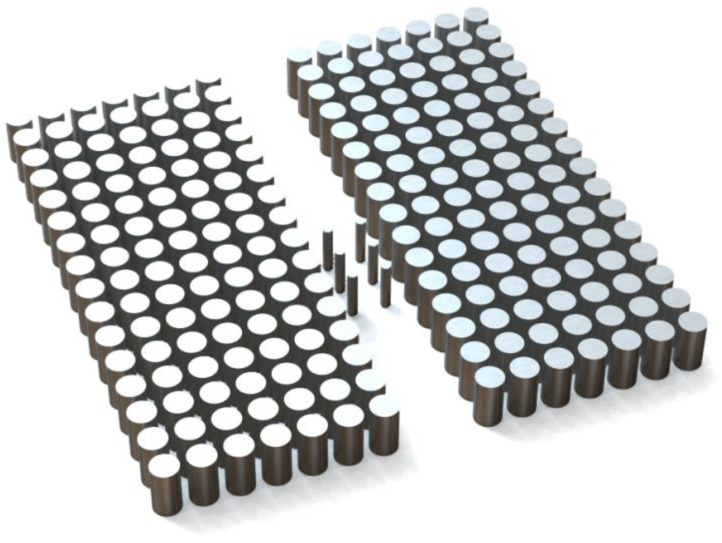
A stereoscopic drawing of the polarization isolator.

**Figure 2:**
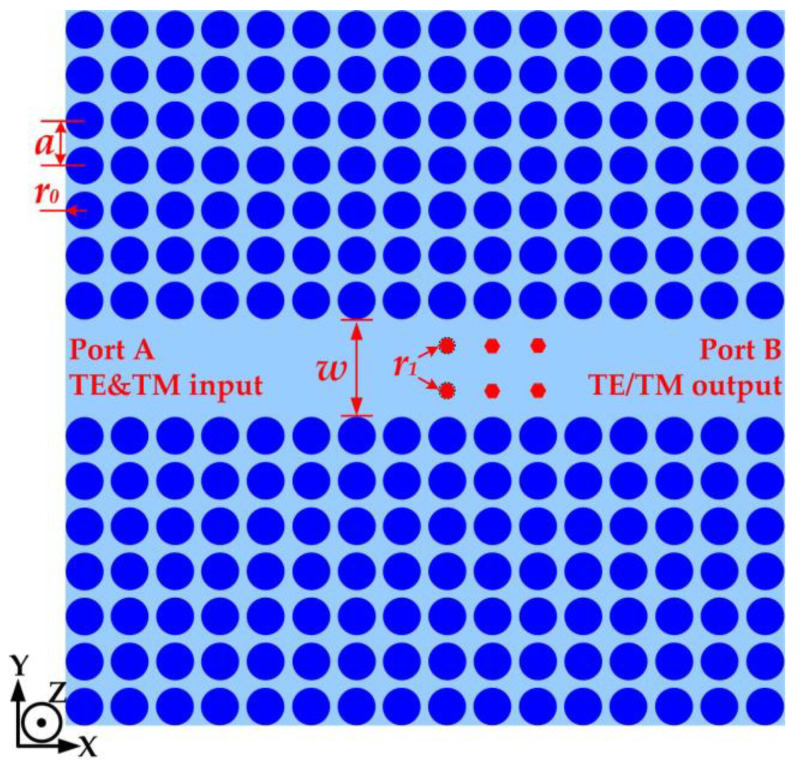
The detailed structural parameters of the polarization isolator in the planform view.

The stereoscopic diagram of the polarization isolator is formed by inserting six smaller hexagonal tellurium rods into the photonic crystal waveguide (PCW), as shown in Figure 1. The PCW consists of two 7 × 16 square lattice tellurium rod arrays with larger radii. All the tellurium rods in Figure 1 are the same material, an orthorhombic silver-white crystal, which is similar to antimony and belongs to an anisotropic medium. The detailed structural parameters of the PhC isolator are shown in Figure 2 below.

Figure 2 shows the detailed structural parameters of the polarization isolator in the planform view. The lattice constant *a* of the square lattice rod array is set at 538 μm for the given central frequency of 0.22 THz. The radius *r*_0_ of the larger rods is 0.4*a* depending on the optimal width of the complete PBG. The straight PCW with the width *w* that is enclosed in two 7 × 16 square lattice tellurium rod arrays is shown in the color blue. The six hexagonal tellurium rods (marked red) with smaller circumradii of *r*_1_ are introduced into the PCW. The two ports of the straight PCW marked Port A and Port B are the input and output ports of the polarization isolator. When the THz waves with the TE and TM modes are incoming from Port A, due to the six rods’ specific isolation on one of the two polarization states, there is only one mode of output at Port B. The PCW works as a THz transmission line so that its transmission efficiency can be optimized by precisely adjusting the width *w*. The optimal value for the height of the rods is generally half of the width. The optimization method has been reported in our previous work [28] and has not been repeated here. Only THz signals within a certain frequency domain can transmit in the PCW. The certain frequency domain relies on the complete PBG of the tellurium PhCs, which is calculated in the next section.

## 3. Analytical and Numerical Results

### 3.1. The Complete PBG of the Tellurium PhCs

The existing research has shown that 2-D square lattice isotropic cylindrical PhCs have no complete PBGs, but studies on anisotropic square lattice tellurium PhCs show that a large complete band gap can be adjusted due to the different levels of permittivity in the Z direction and the X-Y plane [29]. The anisotropy of the tellurium crystal depends on the permittivity tensor $\left[\varepsilon_{r}\right]$, which can be expressed as

(1) $$\left[\varepsilon_{r}\right]=\left[\begin{array}{ccc} espTo & 0 & 0\\ 0 & espTo & 0\\ 0 & 0 & espTe \end{array}\right],$$

where the diagonal elements *espTo* and *espTe* are about 23.04 and 38.44.

**Figure 3:**
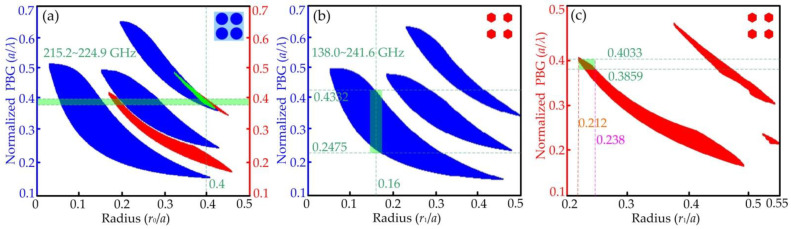
The PBGs of the square lattice tellurium PhCs with increasing radii: (**a**) the 7 × 16 square lattice tellurium rod array; (**b**) the PBGs of the six hexagonal tellurium array for the TE mode; (**c**) the PBGs of the six hexagonal tellurium array for the TM mode.

The PBGs of TE and TM modes are computed by using Bandsolve of Rsoftwave with the rods’ increasing radii, as shown in Figure 3. It can be seen that there are three TE band gaps marked in blue distributed in the normalized frequency domain of about 0.1(*a*/*λ*) to 0.7(*a*/*λ*) for TE modes in the 7 × 16 square lattice tellurium rod array, and they both change regularly with an increasing radius *r*_0_ from 0.05*a* to 0.45*a* in Figure 3a. Overall, the three PBGs are first widening and then narrowing; in addition, the first PBG with a smaller radius is the widest one. The other two narrower gaps are according to lager radii. Compared with the large PBG of the TE mode, the TM band gaps are relatively narrow, marked in red, distributed in the normalized frequency range of about 0.2(*a*/*λ*) to 0.5(*a*/*λ*), as also shown in Figure 3a. Similarly, the two PBGs of the TM mode have the same changing rule as that of the TE mode.

Through careful observation, it is obvious that the TE and TM gaps appear overlapped and the overlapping area marked in green is the complete PBG. The data show that the normalized frequency range of the complete PBG is from 0.3859(*a*/*λ*) to 0.4033(*a*/*λ*), corresponding to the frequency domain from 215.2 to 224.9 GHz, when the tellurium rods’ radii are 0.4*a* with the lattice constant *a*. The width of the PBGs for the TE and TM modes both rely on the ratio of *r*_0_/*a*. A complete PBG can be get as wide as possible by precisely adjusting this ratio, rendering it able to adapt to the requirements of broadband for modern communication equipment.

The PBGs of the six hexagonal tellurium array for the TE mode are shown in Figure 3b, in which there are also three gaps with increasing circumradii *r*_1_ which also have the same changing rule as that in Figure 3a. When the ratio *r*_1_/*a* is 0.16, the normalized frequency range of the PBG for the TE mode covers 0.2475(*a*/*λ*) to 0.4332(*a*/*λ*), corresponding to the frequency domain from 138.0 to 241.6 GHz, which completely covers the frequency domain from 215.2 to 224.9 GHz shown above in Figure 3a. The data show that the six hexagonal tellurium rods can arbitrarily resist the TE waves within the frequency domain of the complete PBG in the PCW. Through observing the PBGs of the six hexagonal tellurium rod array for the TM mode in Figure 3c, the range of minimum values for circumradius *r*_1_ is from 0.212*a* to 0.238*a*, which can make the TM PBG touch the area from 0.3859(*a*/*λ*) to 0.4033(*a*/*λ*). Therefore, the six hexagonal tellurium rods will turn a blind eye to the TM waves when the ratio *r*_1_/*a* is 0.16.

### 3.2. The Transmission Characteristics of the PCW

**Figure 4:**
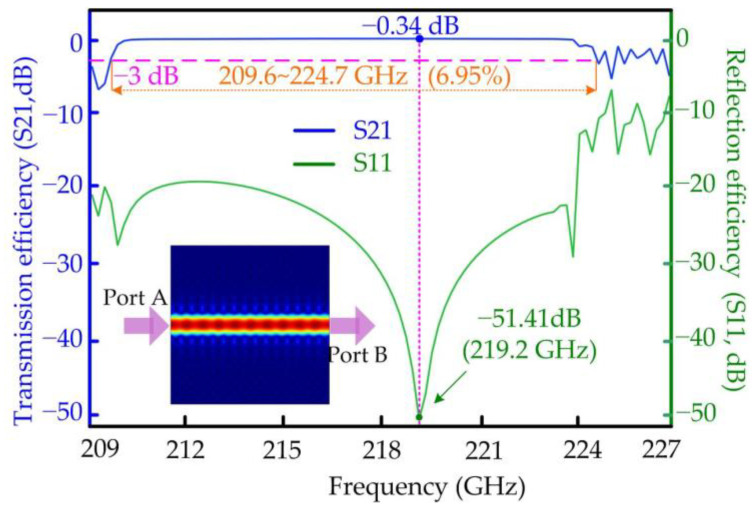
The external characteristics of the PhC waveguide with the increasing frequency for the TE mode.

**Figure 5:**
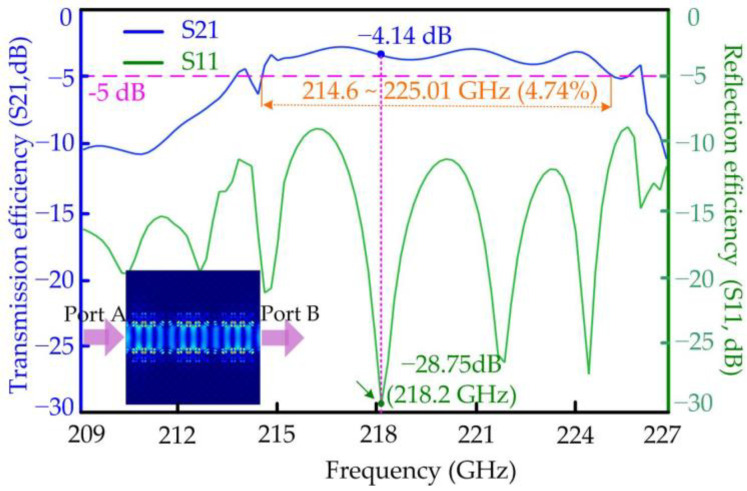
The external characteristics of the PhC waveguide with the increasing frequency for the TM mode.

The transmission characteristics of the PCW and the function of the polarization isolator below are calculated and simulated through the FEM according to the equation of

(2) $$\nabla \times {\mu_{r}}^{-1}\left(\nabla \times \vec{E}\right)-k_{0}^{2}\left(\left[\varepsilon_{r}\right]-\frac{j\sigma }{\omega \varepsilon_{0}}\right)\vec{E}=0,$$

where $\left[\varepsilon_{r}\right]$ is the permittivity tensor expressed in Formula 1, while ω is the angular frequency of the THz signal, the *j* and *σ* are the ampere density and conductivity. $\mu_{r}$ and $\vec{E}$ are the relative permeability and electric intensity vector, respectively. With these structural numbers of *a* = 538 μm, *r*_0_ = 0.4*a*, *r*_1_ = 0.4 *r*_0_ and *w* = 1.2 mm, the transmission characteristics of the designed PhC waveguide will be numerically investigated with the increasing frequency for both the TE and TM modes in this section, as shown in Figure 4 and Figure 5. Here, there are no six smaller tellurium rods in the whole straight PCW. In communications engineering, the S parameters are important performance indexes for communication devices, which are the logarithmic values of the power ratio between the input and output ports. For two-port communication networks, here, the input and output power are checked at Port A and Port B, respectively. The transmission (S21) and reflection (S11) efficiencies of the PCW are calculated with the frequency domain from 209 to 227 GHz, and the curves for the TE and TM modes are drawn in Figure 4 and Figure 5.

The numerical results show that the transmission efficiency of the PCW for the TE mode achieves the maximum value of −0.34 dB at the central frequency of 219.2 GHz and keeps above −3 dB from 209.6 to 224.7 GHz, obtaining a broad relative bandwidth of 6.95 percent, as shown in Figure 4. Meanwhile, the lowest reflection efficiency of the designed waveguide for the TE mode is −51.41 dB, also at the central frequency of 219.2 GHz. The operating bandwidth of the tellurium PCW for the TM mode is narrower than that of the TE mode, whose relative bandwidth is about 4.74 percent around 0.22 THz and above −5 dB, as shown in Figure 5. The lowest reflection efficiency of the designed waveguide for the TM mode is −28.75 dB at the frequency 218.2 GHz. It is observed that there is a co-ownership operating frequency domain from 214.6 to 224.7 GHz for the transmission efficiencies of the TE and TM modes. The THz signals are input from Port A, then transmit steadily in the waveguide, and finally output at Port B both for the TE and TM modes, as shown in the inserted drawings of power distribution in the bottom left of Figure 4 and Figure 5.

### 3.3. The Function of the Polarization Isolator

**Figure 6:**
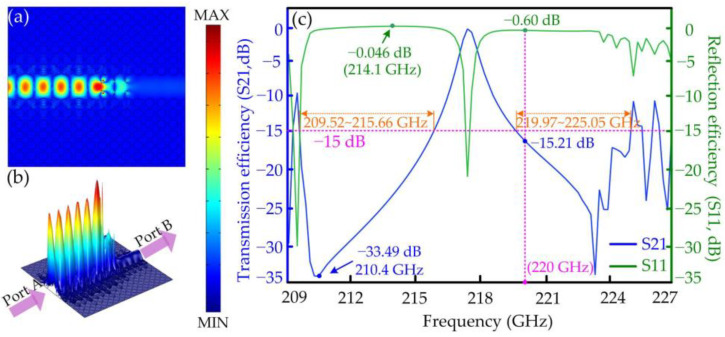
The function and external characteristics of the designed PhCs polarization isolator: (**a**) the planar view of the power distribution of the TE wave; (**b**) the altitudinal view of the power distribution of the TE wave; (**c**) the transmission and reflection efficiencies of the isolator with the increasing frequency.

**Figure 7:**
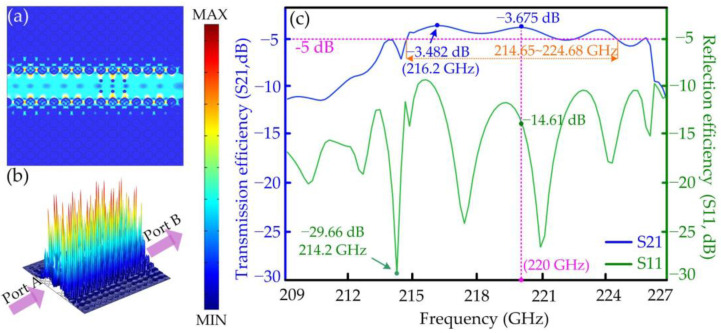
The function and external characteristics of the designed PhC polarization isolator: (**a**) the planar view of the power distribution of the TM wave; (**b**) the altitudinal view of the power distribution of the TM wave; (**c**) the transmission and reflection efficiencies of the isolator with the increasing frequency.

The function and the external characteristics of the polarization isolator are also simulated by the FEM, and the numerical results are shown in Figure 6 and Figure 7. The power distributions of the TE waves in the polarization isolator are simulated at certain frequencies, as shown in Figure 6, in which the planar and altitudinal views of power distributions are shown in Figure 6a and Figure 6b, respectively. At the central frequency of 220 GHz, the TE wave incoming from Port A is almost isolated by the six tellurium rods, resulting in no energy reaching Port B. The curves of the transmission and reflection efficiencies of the isolator with the increasing frequency from 209 to 227 GHz are shown in Figure 6c. The data suggest that the transmission and reflection efficiencies reach the values of −15.21 dB and −0.6 dB, respectively, at the frequency of 220 GHz. The highest isolation of −33.49 dB is achieved at 210.4 GHz, and the maximum reflection efficiency of −0.046 dB (98.95 percent) is achieved at 214.1 GHz. The designed isolator has two operating frequency domains from 209.52 to 215.66 GHz and from 219.97 to 225.05 GHz, in which the isolation keeps above −15 dB. The numerical results shown that the designed polarization isolator has excellent performance when isolating the TE waves.

Conversely, the six hexagonal tellurium rods have almost no effect on the TM wave, which can be confirmed in the results shown in Figure 7. Similarly, the planar and altitudinal views of the power distributions of the TM wave at the interesting frequency of 220 GHz are shown in Figure 7a,b. The TM wave can pass through the six hexagonal tellurium rods smoothly. The transmission and reflection efficiencies of the isolator are calculated with the increasing frequency from 209 to 227 GHz, as shown in Figure 7c. The data suggest that the transmission and reflection efficiencies reach the values of −14.61 dB and −3.675 dB, respectively, at the frequency of 220 GHz. The highest transmission efficiency of −3.482 dB is achieved at 216.2 GHz, and the lowest reflection efficiency of −29.66 dB is achieved at 214.2 GHz. Compared with the curves of the PCW for the TM mode in Figure 5, the trend of the transmission and reflection efficiencies is basically same with the changing frequency between the PCW and the PhC isolator. The experimental results in [27] reported a remarkable Faraday rotation angle of 45°, and the THz isolator achieved an isolation of 23 dB around the 1.2 THz band.

To sum up, the numerical results show that the designed polarization isolator has excellent performance when isolating TE waves but turns a blind eye to TM waves. The function of the polarization isolator can also be understood to perfectly separate the TM mode from the mixed modes. The feasible approach of the structure’s fabrication can be divided into two steps: the first step is making a precise mold; the second step is fixing the polished rods on the mold to form an array structure.

## 4. Conclusions

In this work, we focus on designing a novel THz polarization isolator by inserting six smaller hexagonal tellurium rods into a fully polarized PCW. The waveguide with high efficiency of −0.34 dB consists of two 7 × 16 2-D square lattice tellurium rod arrays. The TE and TM photonic band gaps of the tellurium photonic crystals are calculated by the plane wave expansion method, which happen to coincide at the normalized frequency domain from 0.3859(*a*/*λ*) to 0.4033(*a*/*λ*), corresponding to the frequency domain from 0.2152 to 0.2249 THz. The operating bandwidth of the tellurium photonic crystal waveguide covers 0.2146 to 0.2247 THz, calculated by the finite element method, which fits perfectly with the complete PBG. The six hexagonal tellurium rods with smaller circumradii of 0.16*a* can effectively isolate the TE waves and turn a blind eye to the TM waves. The polarization isolation also achieves excellent isolation of −33.49 dB at the central frequency of 0.2104 THz and the optimal reflection efficiency of 98.95 percent at the frequency of 0.2141 THz. The designed isolator with a unique function and high performance provides a promising approach for implementing fully polarized THz devices in future 6G communication systems. Meanwhile, ultra-fast THz generation based on dielectric metasurfaces [30,31] and silicon photonic platforms [32] also has important research value in the field of THz communication.

## Data Availability

Data is contained within the article.
